# Expansive reed populations—alien invasion or disturbed wetlands?

**DOI:** 10.1093/aobpla/ply014

**Published:** 2018-02-23

**Authors:** Kim Canavan, Iain D Paterson, Carla Lambertini, Martin P Hill

**Affiliations:** 1 Department of Entomology and Zoology, Rhodes University, Grahamstown, South Africa; 2 Department of Agricultural Science, University of Bologna, Viale Fanin, Bologna, Italy

**Keywords:** Hybridization, *Phragmites*, *australis*, *Phragmites mauritianus*, phylogeography, reed expansion, tall-statured grasses

## Abstract

The tall-statured grasses in the genus *Phragmites* are dominant vegetation in wetlands worldwide and thus play a vital role in ecosystem functioning. As a result, *Phragmites* spp. are some of the most widely studied plants; particularly in areas where changes to their abundances have occurred, most notably in Europe and North America. In southern Africa a pattern of reed expansion has occurred in recent decades that has shown a similar trend to cryptic invasions reported in North America. This study used molecular techniques to explore the phylogeography of *P. australis* and *P. mauritianus* in the region to investigate whether the expansion is due to an alien invasion or local factors such as wetland disturbance. Three haplotypes were found and all haplotypes are presently considered African haplotypes (haplotype K for *P. australis* and haplotype V and AP for *P. mauritianus*). Both *Phragmites* spp. were found to have high genetic diversity. Microsatellite and *grass-waxy* analysis also found evidence of hybridization between the two species. No evidence was found for a recent cryptic invasion of non-native haplotypes in southern Africa. The expansion of *P. australis* and *P. mauritianus* is therefore most likely a result of anthropogenic activity. Identifying and mitigating the human-mediated factors that may be contributing to reed growth, such as eutrophication and sedimentation, should be the focus of future management protocols.

## Teaser

In southern Africa a pattern of *Phragmites* spp. reed expansion has occurred in recent decades that has shown a similar trend to cryptic invasions reported in North and South America. This study used molecular techniques to explore the phylogeography of *P. australis* and *P. mauritianus* and found no evidence of cryptic invasion. The expansion of *P. australis* and *P. mauritianus* is therefore occurring within native haplotypes and is most likely a result of anthropogenic activity that has disturbed wetlands.

## Introduction

Wetlands are considered one of the most valuable ecosystems as they are areas of naturally high species diversity, a diverse array of biological and physical processes, provide critical habitat structure and improve water quality (Cronk and Fennessy 2016). It is the dominant plants within these ecosystems that are most influential and as such, the greatest functional changes occur if the abundances of these species change (Richardson *et al.* 2007). Species in the genus *Phragmites* are dominant wetland plants worldwide. There are four species in the genus including *P. australis*, *P. mauritianus*, *P. japonicus* and *P. karka* (Clevering and Lissner 1999). The species are all morphologically similar and presumed to be closely related (Lambertini *et al.* 2006). The abundance and distribution of these *Phragmites* spp. has been found to have important consequence for wetland ecosystem functioning.

The most widely distributed and abundant *Phragmites* species is *P. australis*, which is also one of the most studied plants in the world (Pyšek *et al.* 2008). A large amount of research on the plant’s ecophysiology and population dynamics has focused on North American and European populations, primarily due to these regions having had recent changes to *P. australis* abundance and distribution (Meyerson *et al.* 2016). In Europe, *P. australis* has been the focus of intense research since the 1970s in response to declining reed beds (Tscharntke 1992; Brix 1999). In North America, on the other hand, there have been cryptic invasions of *P. australis* that have resulted in rapid expansions of reed populations (Saltonstall 2002; Lambertini *et al.* 2012a). Such cryptic invasions occurred when non-native haplotypes from Europe were introduced to North America and were able to outcompete native haplotypes (Saltonstall 2002). Since then, the expansion of certain reed populations in other regions has also been attributed to cryptic invasion including parts of Canada (Lelong *et al.* 2007) and South America (Guo *et al.* 2013). Such phylogeographic work is however under-represented in Africa (Lambertini *et al.* 2012b).

The three species of *Phragmites* which occur in Africa are *P. australis*, *P. mauritianus* and *P. karka* (syn. *P. vallatoria*) (Gordon-Gray and Ward 1971; Veldkamp 1994). Despite their importance to wetland ecosystem health, very little is known about any of the species in the region. Yet, in southern Africa where the two species *P. australis* and *P. mauritianus* overlap, there is growing evidence that there is a changing pattern of *Phragmites* spp. abundance and distribution. Although *P. australis* is considered a native species in southern Africa, having been present in the region since the Late Quaternary period according to pollen fossil records (Scott 1982), in recent decades there has been a considerable expansion of the range and abundance of the species in many wetland ecosystems (Weisser and Parsons 1981; Russell 2003; Russell and Kraaij 2008). This expansion is similar to the cryptic invasions in North America whereby reed stands grow to form dense monospecific stands that can negatively impact ecosystem functioning (Chambers *et al.* 1999; Lambert *et al.* 2010; Hazelton *et al.* 2014). South African wetland areas have particularly high diversity, as the region is ecologically diverse with nine terrestrial biomes that have a rich flora and high levels of endemism (Cowling and Hilton-Taylor 1997; Rutherford *et al.* 2006). However, due to anthropogenic activities these areas are increasingly becoming disturbed and are now currently considered the most threatened ecosystem type (Driver *et al.* 2011). As such it is important to address the potential for cryptic invasions of non-native haplotypes in the country (Canavan *et al.* 2014).

In considering the potential for cryptic invasions by non-native *P. australis* genotypes, *Phragmites mauritianus* can serve for comparisons of genetic diversity levels because it is endemic to Africa and therefore native (Gordon-Gray and Ward 1971). Genetic variation is generally agreed to be structured in space and time (Loveless and Hamrick 1984); with higher levels of genetic diversity being found in plants in their native range (Dlugosch and Parker 2008). To date, *P. mauritianus* remains a poorly studied species where at present there has been no in-depth investigation of its genetic diversity and dispersal mechanisms.

*Phragmites australis* and *P. mauritianus* are closely related and share the same reproductive strategy and plant architecture. Differentiating the two species is difficult; Gordon-Gray and Ward (1971) characterizes *P. mauritianus* from *P. australis* by having lax mature inflorescence with drooping branches, bare internodes with exposed axillary buds after maturation, well-branched stems and stiffer pointed leaves. Both *P. australis* and *P. mauritianus* can reproduce both sexually and asexually (Fanshawe 1972; Ailstock and Center 2000; Saltonstall *et al.* 2010). Sexual reproduction involves the production of anemochorous seeds from flower panicles; for both reeds seed viability is highly variable (Fanshawe 1972; McKee and Richards 1996; Ailstock and Center 2000). For *P. australis*, seeds do not remain viable for long; however, it has been found that where germination occurs the density of germinated seeds is almost as high as the number of viable seeds produced (~700 seeds per m^2^) (Baldwin *et al.* 2010; Hazelton *et al.* 2014). Vegetative reproduction is the primary mode of reproduction with spread occurring from a root system known as a rhizosphere (Fanshawe 1972; Hellings and Gallagher 1992; Clevering and Lissner 1999). Hybridization between *P. australis* and *P. mauritianus* was thought to be impossible due to differences in chromosome number (Lambertini *et al.* 2006): *Phragmites mauritianus* is tetraploid while *P. australis* is octoploid in southern Africa (Gordon-Gray and Ward 1971). Yet with improved molecular markers, evidence of hybridization among *Phragmites* taxa with different ploidy levels has been uncovered (Chu *et al.* 2011; Lambertini *et al.* 2012a; Meyerson *et al.* 2012; Lambertini 2016).

In this study, we investigated the phylogeography of *P. australis* and *P. mauritianus* in southern Africa. Our aim was to trace, or rule out, a potentially cryptic invasion of *P. australis* in southern Africa, and explore the management of reed beds in wetland areas in light of population dynamics.

## Methods

### Study sites and DNA extraction

**Figure 1.**
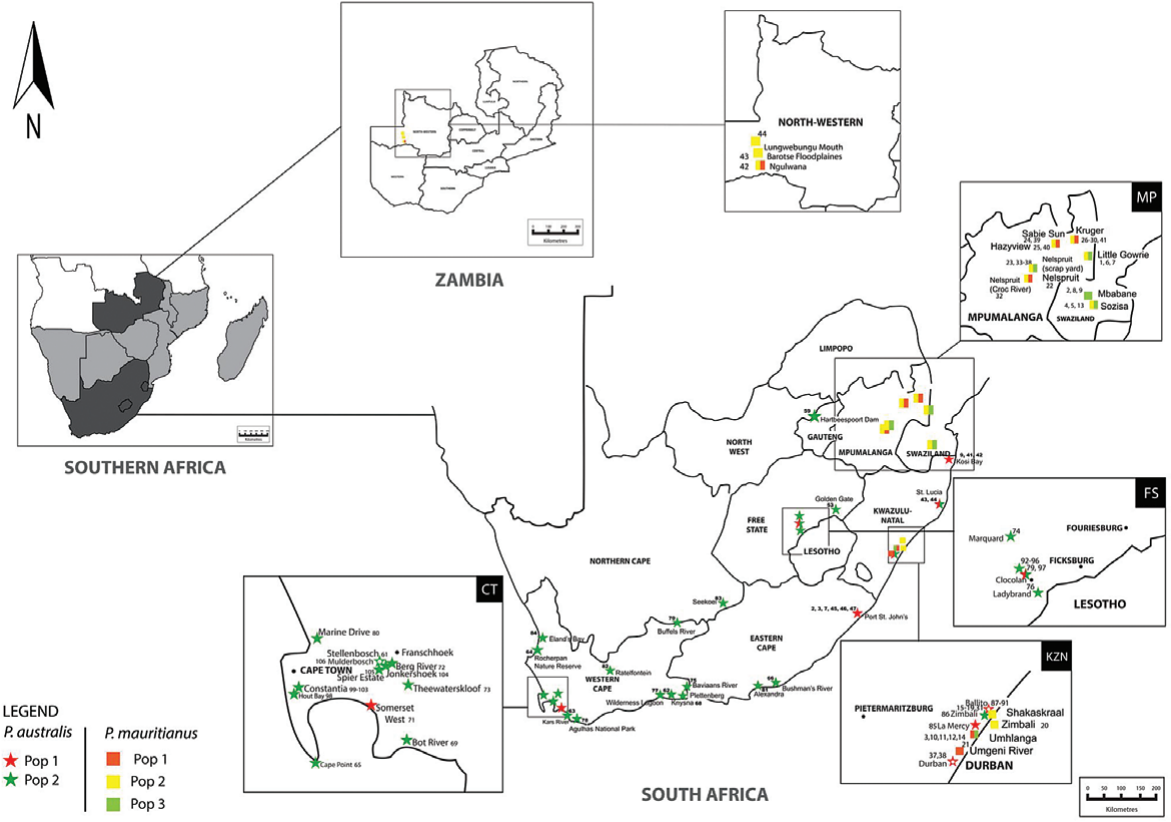
Map of southern Africa showing *P. australis* and *P. mauritianus* sampling sites. Sampling sites for *P. australis* are indicated by a star symbol along with the sample number **[see****Supporting Information—Table S1****]**. Sampling sites for *P. mauritianus* are indicated by a square symbol along with the sample number **[see****Supporting Information—Table S2****]**. Populations found for both reed species are shown (*P. australis*: pop 1—red, pop 2 – green; *P. mauritianus*: pop 1—orange, pop 2—yellow, pop 3—green), refer to populations inferred by the Evanno method and based on Bayesian clustering analysis of four microsatellite loci with STRUCTURE (Pritchard *et al.* 2000).

Fresh leaf samples of both *P. australis* and *P. mauritianus* (Fig. 1) were collected from sites in southern Africa **[see**Supporting Information—Table S1 and S2**]**. For *P. australis*, 39 sites were sampled from wetlands across South African’s nine biomes, namely the Albany thicket, grassland, savanna, Nama-Karoo, forest, fynbos, desert, Indian Ocean Coastal Belt and thicket biomes (Rutherford *et al.* 2006). For *P. mauritianus*, 16 sites were sampled from wetlands within the reed’s distribution which is restricted to tropical Africa (Gordon-Gray and Ward 1971) including the Indian Ocean Coastal Belt biome in northeastern South Africa, the subtropical moist forest biome in Swaziland (Rutherford *et al.* 2006) and the Zambezian cryptosepalum dry forest ecoregion in Zambia (Fig. 1) (Schulze and McGee 1978). The distribution of *P. australis* and *P. mauritianus* was only found to overlap in the KwaZulu-Natal province in South Africa. For both species, a number of populations were chosen at random to take more samples from, to give an indication of intra-site variability. DNA was isolated from samples with the Qiagen DNeasy Plant Mini Kit (Qiagen Inc., Valencia, CA, USA) as described in Lambertini *et al.* (2006) from young leaves stored in silica gel, after leaves were crushed using liquid nitrogen.

### Sequences

Two non-coding regions in the chloroplast genome, *trn*T–*trn*L and *rbc*L–*psa*I, were amplified (Saltonstall 2002). Ten picomoles of forward and reverse primers were added to 12.5 μL of Promega MasterMix (Madison, WI, USA) (reaction concentration of 1 U of *Taq*, 1.5 mM MgCl_2_ and 0.2 μM dNTPS), 2 μL of Promega magnesium chloride and 7 μL of template DNA per reaction. Promega nuclease-free water was added to reach a final volume of 25 μL. Amplification was run in one of the following machines: Labnet Multigene II (Labnet, Edison, NJ, USA) or Applied Biosystems 2720 thermal cycler (Applied Biosystems, Foster City, CA, USA). For the *trnL*b region, the PCR cycling protocol was 94 °C for 1 min, 35 cycles of 94 °C for 1 min, 56 °C for 1 min, 72 °C for 2 min, followed by a final extension at 72°C for 5 min. For the *rbc*L region, the same cycling protocol was used; however, the annealing temperature was lowered to 45 °C.

PCR products were sent to Stellenbosch University, Stellenbosch, South Africa, or to Inqaba Biotec, Johannesburg, South Africa, for sequencing, using a BigDye Terminator ver. 3.1 Cycle Sequencing Kit (Applied Biosystems, Foster City, CA, USA). Capillary electrophoresis was done using an ABI 3100 genetic analyser (Applied Biosystems, Foster City, CA, USA) at Stellenbosch University or using an ABI 3500 genetic analyser (Applied Biosystems, Foster City, CA, USA) at Inqaba Biotec.

### Microsatellites

From Saltonstall (2003), four primers (PaGT4, PaGT8, PaGT9 and PaGT22) were selected due to their high variability in the sample set. Primers were fluorescently labelled by Applied Biosystems Inc., UK; PaGT4, PaGT8 and PaGT22 with 6-FAM dye and PaGT9 with NED dye using standard FA parameters (DS-33 Matrix Standard G5 dye set). Eighteen microlitres of Promega Master Mix (Madison, WI, USA), 10 pmol forward and reverse primers, 3 μL of template DNA and Promega nuclease-free water were added to reach a volume of 20 μL. The PCR cycling protocol was 94 °C for 12 min, followed by 35 cycles of 94 °C for 30 s, 50–56 °C for 30 s, 72°C for 40 s and an extension of 72 °C for 5 min. PCR products were diluted 20× with sterile water prior to capillary electrophoresis (sent to Stellenbosch University, Stellenbosch, South Africa or to the Inqaba Biotec lab, Johannesburg, South Africa) using either an ABI 3100 genetic analyser (Applied Biosystems, Foster City, CA, USA) or an ABI 3500 genetic analyser (Applied Biosystems, Foster City, CA, USA).

An error rate was determined by replicating 10 % of the samples. To further test the accuracy of the results, the same samples were run in different labs. To avoid subjectivity in scoring of peaks, any peaks that were ambiguous and in particular with stutter peaks were scored as missing data. The resulting error rate was 15.22 % and was found due to both false allele amplification and allelic dropout with both factors equally contributing to the error rate. The relatively high error rate is most likely a result of the fact that the duplicated samples were run on different ABI machines. All potential hybrids were amplified twice to ensure reproducibility of the results. The replication of hybrids had an error rate of 0 %.

### *Grass-waxy* analysis

Two DNA fragments amplified by the *grass-waxy* primers designed by Mason-Gamer *et al.* (1998) were used as diagnostic DNA fragments for *P. mauritianus* and Mediterranean *P. australis* by Lambertini *et al.* (2012b). We designed specific primers **[see****Supporting Information—Table S3****]** for the two fragments and amplified all our samples. One microlitre of template DNA was added to 10 μL 2× Mastermix (VWR Amplicon), 10 pmol of forward and reverse primers, and sterile water to reach a total volume of 20 μL. The cycling protocol was 94 °C for 3 min, 40 cycles of 94 °C for 30 s, 62 °C for 40 s, 72 °C for 40 s, followed by 72 °C for 7 min. Products were run in a 1.5 % agarose gel for 1 h 30 min at 125 V, 84 mA and stained with ethidium bromide.

The presence or absence of the resulting bands of 100 bp for *P. mauritianus* and 200 bp for Mediterranean *P. australis* was recorded and the PCR product was then sequenced at the Inqaba Biotec lab, Johannesburg, South Africa using an ABI 3500 genetic analyser (Applied Biosystems, Foster City, CA, USA).

### Sequence data analysis

Sequences were assembled and manually edited in GeneStudio ver. 2.2.0.0 (GeneStudio, Inc.). Alignment of sequences was done in MEGA ver. 5.2.2 including all worldwide haplotypes downloaded from GenBank using ClustalW set to default parameters (Kumar *et al.* 2012).

### Microsatellite data analysis

Chromatogram alignment and allele sizing was done using Geneious ver. 8.1.7 (Kearse *et al.* 2012). Given the polysomic nature of the sample set (more than two alleles per locus), the data set was entered into a binary matrix (1 = presence, 0 = absence of homologous alleles) and analysed using GenAIEx ver. 6.5 (Peakall and Smouse 2012). Pairwise genetic distances were calculated based on the number of shared alleles per locus (Euclidean distances). The output matrix of genetic distances was then used to run a principal coordinates analysis (PCoA). Genetic diversity was compared between *P. mauritianus* and *P. australis* by calculating Nei’s unbiased genetic identities (Nei 1973) and Shannon information index (Lewontin 1995) and number of effective alleles (*N*_e_) with the program PopGene ver. 1.32 population genetic analysis (Yeh and Boyle 1997) as done in previous studies (Lambertini *et al.* 2012b). To compare the allelic diversity to worldwide lineages in Saltonstall (2003), the average total number of alleles (*A*_o_) were recorded across the four loci and observed heterozygosity (*H*_o_) was measured for the two species as the percentage of heterozygotes (i.e. genotypes with more than one allele at a locus) across the four loci **[see****Supporting Information—Table S4****]**.

The samples were initially classified as either *P. australis* or *P. mauritianus* based on their cpDNA matrilineages (*trn*T–*trn*L and *rbc*L–*psa*I). The nuclear ancestry of the two populations was subsequently tested based on the four microsatellite loci using a Bayesian genetic clustering algorithm implemented in STRUCTURE version 2.3.4 (Pritchard *et al.* 2000). An admixture model was used that assumed independent allele frequencies with 10 iterations for each run. Each run consisted of 1000000 MCMC steps and a burn-in period of 100000. The number of populations (*K*) was tested from 1 to 10 and *K* was inferred with STRUCTURE HARVESTER (Earl and vonHoldt 2012) following Evanno *et al.* (2005). Models were also run including only *P. australis* or *P. mauritianus* samples according to the same parameters.

## Results

### cpDNA

All *P. australis* samples (*n* = 61) were cpDNA haplotype K (Saltonstall 2002), which has also been found elsewhere in Africa (Saltonstall 2002; Lambertini *et al.* 2012a) and in isolated occurrences in Europe (Spain) (Lambertini *et al.* 2012b). *Phragmites mauritianus* had two haplotypes; all South African samples (*n* = 41) were found to be haplotype V, while the samples from Zambia were haplotype V (*n* = 1) and haplotype AP (*n* = 2) (Lambertini *et al.* 2012b). Haplotype V was previously found in the *P. mauritianus* population in South Africa and in one sample from Equatorial Guinea labelled as *P. vallatoria* (syn. *P. karka*), whereas haplotype AP was found only in *P. mauritianus* samples from Zambia (Lambertini *et al.* 2012b).

### Microsatellites

A total of 36 alleles were found in *P. australis* samples and 27 alleles for *P. mauritianus* samples across all four loci **[see****Supporting Information—Table S4****]**. The number of alleles amplified in each sample varied for *P. australis* and *P. mauritianus*; *P. australis* samples had an average of 2.16 ± 0.94 alleles amplified compared to only 1.56 ± 0.71 amplified for *P. mauritianus.* For *P. australis*, a maximum of four alleles were found; however, *P. mauritianus* had a maximum of only two alleles. *Phragmites australis* was found to have a higher average of total number of alleles (*A*_0_ = 9 ± 2.83) found at each loci compared to the same loci in worldwide samples from North America and Europe from Saltonstall (2003). However, *P. mauritianus* had a similar average number of total alleles found in the four loci (*A*_0_ = 6.75 ± 1.26) compared to European introduced populations in North America (*A*_0_ = 7 ± 3.56) and native populations in Europe (*A*_0_ = 8.5 ± 4.2) **[see****Supporting Information—Table S5****]**.

**Figure 2.**
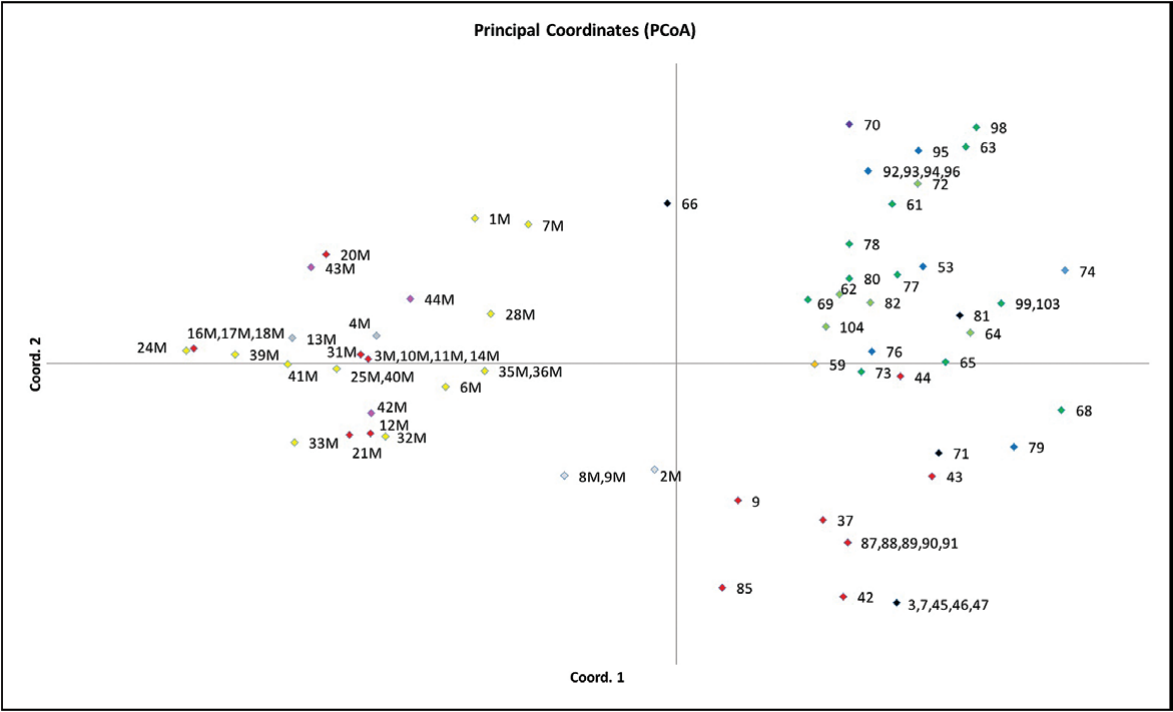
Principal coordinates analysis (PCoA) built on Euclidean genetic distances between *P. australis* and *P. mauritianus* samples in Southern Africa using PaGT4, PaGT8, PaGT9 and PaGT22 microsatellite primers. Samples with missing data were excluded. Coordinate 1 accounts for 22.55 % and Coordinate 2 accounts for 12.25 % of the variation. Colours represent geographic area defined by sampling region in Southern Africa; red—KwaZulu-Natal, blue—Free State, yellow—Mpumalanga, black—Eastern Cape, green—Western Cape, orange—North West, purple—Northern Cape, pink—Zambia and grey—Swaziland. *Phragmites mauritianus* samples all clustered to the left axis and *P. australis* samples clustered to the right axis. Samples followed by the letter M denote *P. mauritianus* samples and samples with no letters denote *P. australis* samples. Four samples (66, 2M, 8M, 9M) were found to cluster halfway between the two groups.

**Figure 3.**
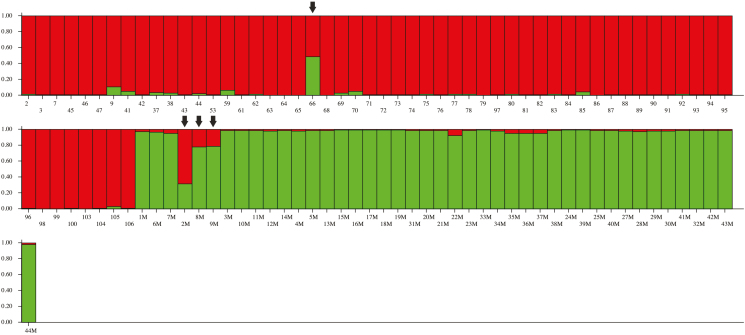
Genetic population structure of *Phragmites australis* and *Phragmites mauritianus* from samples in southern Africa, based on Bayesian clustering analysis of four microsatellite loci with STRUCTURE (Pritchard *et al.* 2000). According to the Evanno method, two populations were inferred **[see****Supporting Information—Fig. S1****]**. Based on cpDNA matrilineage, the red cluster corresponds to *P. australis* individuals, whereas the green cluster represents *P. mauritianus*. The values in the y-axis display the shared ancestry according to percentage membership into *P. australis* or *P. mauritianus* populations **[see****Supporting Information—Tables S1** and **S2****]**. The downward arrows point to samples with shared alleles with the other species (in order from top left, samples 66, 2M, 8M, 9M).

The PCoA separated the two species into two distinct groups but four samples (66, 2M, 8M, 9M) were not found to group with either of the two species and instead clustered halfway between the *P. australis* and *P. mauritianus* groups (Fig. 2). According to STRUCTURE HARVESTER analysis, *P. australis* and *P. mauritianus* samples have two ancestral populations (*k* = 2) and STRUCTURE was then used to cluster the samples into the two groups (Fig. 3) and also **see****Supporting Information—Fig. S1** . Four samples both within the *P. australis* and *P. mauritianus* clusters had a proportion of their genotype which shares ancestry with the other species. For *P. australis*, sample 66 had a signature of shared ancestry, with 51.8 % membership in the *P. mauritianus* population and 48.2 % membership in the *P. australis* population. The sample shared three alleles with *P. mauritianus* samples (loci: PaGT4—allele 270; PaGT9—alleles 196 and 202). For *P. mauritianus*, sample 2M had 67.2 % membership in the *P. australis* population and 32.8 % membership in the *P. mauritianus* population, whereas samples 8M and 9M had a higher membership in the *P. mauritianus* population than in that of *P. australis*. These samples represent two allelic genotypes (samples 8M and 9M were found to be clones of the same genotype **[see****Supporting Information—Table S2****]**) and together had seven shared alleles with *P. australis* samples (loci: PaGT4—allele 276; PaGT8—allele 175; PaGT9—alleles 198, 200; and PaGT22—alleles 173, 180 (samples 8M and 9M) and allele 183 (sample 2M)). The samples were also found to be intermediate between the two species in the PCoA (Fig. 2), suggesting that hybridization may have occurred.

STRUCTURE HARVESTER determined that *P. australis* samples clustered into two populations and *P. mauritianus* samples clustered into three populations **[see****Supporting Information—Table S1** and **S2****]**. For *P. australis*, the two populations were found to correspond with groupings found in the PCoA. Almost all samples that grouped with Population 1 were found along the coast of South Africa except for one sample (sample 79) (Fig. 1). The remaining samples for Population 2 were found across the range of *P. australis*’ distribution in South Africa. A number of samples from this second group, as well as sample 79 from the Free State province, were found to have an admixed ancestry from the two populations showing the occurrence of gene flow between the two populations in the coast and inland. For *P. mauritianus*, STRUCTURE HARVESTER analysis determined that there are three populations in South Africa and when plotted in STRUCTURE it was found that there is no geographic pattern among these populations (Fig. 1 and **see****Supporting Information—Table S2**).

For *P. australis*, two stands sampled in Kosi Bay (samples 9, 41, 42) and St. Lucia (samples 43, 44) were found to have different genotypes **[see****Supporting Information—Table S1****]**. For *P. mauritianus*, only two stands did not have variation and most had different genotypes **[see****Supporting Information—Table S2****]**. *Phragmites mauritianus* was also found to have a higher Shannon information index (*I*) (0.425 ± 0.204), number of effective alleles (*N*_e_) (1.446 ± 0.333) and higher Nei’s diversity (*h*) (0.273 ± 0.161) compared to *P. australis* samples (*I* = 0.333 ± 0.231, *N*_e_ = 1.339 ± 0.358, *h* = 0.208 ± 0.177) (Table 1). However, these differences were not statistically significant.

**Table 1.** Comparison of genetic diversity for *P. australis* (Pa) and *P. mauritianus* (Pm) in southern Africa from this study based on four microsatellite loci (over populations for each loci). Sample size (*N*), observed total number of alleles (*N*_a_), number of populations (*N*_pop_), number of effective alleles (*N*_e_), Shannon information index (*I*), Nei’s gene diversity (*h*) and SD. No significant differences were found between *P. australis* and *P. mauritianus* in southern Africa (*P* values obtained with Mann–Whitney *U* test, *P* < 0.050).

	*N*	*N*_a_	*N*_pop_	*N*_e_ (SD)	*I* (SD)	*h* (SD)
Pa—SA	61	36	39	1.339 ± 0.358	0.333 ± 0.231	0.208 ± 0.177
Pm—SA	44	27	16	1.446 ± 0.333	0.425 ± 0.204	0.273 ± 0.161

### *Grass-waxy* bands

A nuclear DNA band of ~200 bp was amplified in 77 % of the *P. australis* samples **[see****Supporting Information—Table S1****]**. This band has previously been found to be exclusive to plants from the Mediterranean region and was used as a marker to trace genotypes of this region (Lambertini *et al.* 2012b). However, 10 *P. australis* samples did not have this band present. The shorter band of ~100 bp, previously recorded to be exclusive to *P. mauritianus* and associated hybrids with *P. australis* (Lambertini *et al.* 2012b) was also found in 27 of the *P. australis* samples and for 10 of these samples was the only band present. For the *P. mauritianus* samples, all reeds were found to have the 100-bp band and none had the 200-bp band **[see****Supporting Information—Table S2****]**.

The sequence of the 200-bp fragment matched the NCBI accession no. JF317300 (Lambertini *et al.* 2012a), previously amplified in samples from the Mediterranean, Senegal sample and the Delta-type in the Gulf Coast of North America. The sequence of the 100-bp fragment aligned with *P. mauritianus* NCBI accession no. JF317301 (Lambertini *et al.* 2012a), and its samples from Uganda, Burkino Faso, the hybrid populations with *P. australis* in Senegal, South America and the Land-type in the Gulf Coast of North America.

## Discussion

Both chloroplast and nuclear markers suggest that *P. australis* and *P. mauritianus* populations should remain being considered native to southern Africa. For both species, the haplotypes found (K, V and AP) are all recognized as native to the region and microsatellite analysis determined relatively high allelic diversity compared to each worldwide lineages from Saltonstall (2003). The population diversity of *P. australis* was found to be similar to the endemic *P. mauritianus* seen through similar Shannon (*I*) and Nei’s diversity (*h*) indices; *P. australis* had a slightly lower diversity although this was not statistically significant. These results suggest that haplotype K populations have a long history in southern Africa and therefore, a cryptic invasion, similar to that found in North America can reasonably be ruled out. The recent expansion of reed beds in southern Africa can most likely be attributed to anthropogenic activities and in such cases *P. australis* can be labelled expansive and not invasive (Pyšek *et al.* 2004).

Previous phylogeographic studies have determined that haplotype K is not restricted to the African continent and has been found in Mediterranean Europe (Lambertini *et al.* 2012b). Many of the southern African samples analysed in this study also carry the diagnostic *waxy* 200-bp band of Mediterranean *Phragmites*, as previously found by Guo *et al.* (2013), who for this reason, included South Africa in the native range of the Mediterranean population. Although our study does not contradict Guo *et al.* (2013), further research including samples from the African continent and the Mediterranean region is needed to assess the centre of origin and distribution range of Mediterranean *Phragmites* across Africa and conclusively shed light on haplotype K and the *waxy* 200 band became present in both European and southern African populations.

Two distinct ancestral *P. australis* populations were identified in southern Africa and such populations appeared independent from either the presence/absence of the *waxy* 200-bp band or having shared alleles with *P. mauritianus*. Population 1 had seven out of eight sites found along the coastline of South Africa while Population 2 had sites from across the range of areas sampled. The distinction of Population 1 could be a result of local differentiation to a coastal niche and could reflect adaptation to ecological heterogeneity or a different ecotype as *P. australis* is known to have halophytic and glycophytic populations (Turesson 1922; Waisel 1972; Takahashi *et al.* 2007; Wu *et al.* 2008; Gao *et al.* 2012). For example, in the Yellow River Delta, China, *P. australis* was found to have population structuring in microsatellite alleles that is believed to be a result of varying soil salinity (Gao *et al.* 2012). In such cases, *P. australis* adapts to different saline conditions by adjusting its plant physiology which results in genetic differentiation over time (Takahashi *et al.* 2007).

For *P. mauritianus*, two haplotypes known as haplotype V and AP were found across all sites. Both haplotypes have been found to be unique to *P. mauritianus* and restricted to the African continent (Lambertini *et al.* 2012a).

Evidence of gene flow between *P. australis* and *P. mauritianus* populations in southern Africa was found in both microsatellite and *grass-waxy* results. Microsatellite markers determined three genotypes (66, 2M, 8M and 9M) in *P. australis* and *P. mauritianus* monospecific populations that had shared alleles. This may be indicative of recent hybridization events between geographically distant populations. Furthermore, *grass-waxy* analysis highlighted more ancient exchanges between the *Phragmites* species. A 100-bp band that has previously been found to be unique to *P. mauritianus* and its hybrid populations (Lambertini *et al.* 2012a, b) was also carried by a number of *P. australis* samples in South Africa. Interestingly, the occurrence of the 100-bp band did not affect the microsatellite structure of the *P. australis* populations. It is therefore likely that the high frequency of this band in the *P. australis* population is due to ancient exchanges of genetic material between the two species that have been conserved. This indicates that as previously hypothesized (Chu *et al.* 2011; Lambertini *et al.* 2012a, b; Meyerson *et al.* 2012) hybridization has taken place in the evolution of the genus *Phragmites*. Within *P. mauritianus* populations there was no occurrence of the 200-bp band unique to *P. australis*; this may indicate that *P. australis* populations are more susceptible to the assimilation of *P. mauritianus* genetic material.

*Phragmites australis* and *P. mauritianus* have remained distinct species in southern Africa despite having an overlapping distribution and showing evidence of hybridization; one explanation is that they have different habitat requirements. Gordon-Gray and Ward (1971) observed in KwaZulu-Natal, South Africa, that *P. mauritianus* prefers areas with permanent, moving water, especially in areas with recent disturbance, whereas *P. australis* is mostly found in areas with restricted drainage that are usually isolated from the main stream. These differences may be either related to different physiological paths in the two species, and/or may be a result of different ploidy levels between the two species in southern Africa. *Phragmites australis* was found to have a higher number of amplified alleles compared to *P. mauritianus* which indicates a higher ploidy level; this agrees with the finding of Gordon-Gray and Ward (1971) who found that South African *P. australis* is octoploid (2*n* = 8*x* = 96) and *P. mauritianus* is a (disomic) tetraploid (2*n* = 4*x* = 48). Octoploids generally have increased plant biomass and tetraploids are generally found to be more opportunistic with high plasticity (te Beest *et al.* 2011).

In addressing the recent expansion of *Phragmites* spp. it is therefore likely that reed encroachment has been facilitated by anthropogenic activities which have disturbed wetlands. Most Southern African wetlands have been heavily degraded, most notably from drainage, eutrophication and input of industrial effluents (Driver *et al.* 2011; Fernandes and Adams 2016; Lemley *et al.* 2017; Sieben 2017). For example, Russell and Kraaij (2008) proposed that *P. australis* encroachment in the Wilderness Lakes, Western Cape, South Africa, was a result of a multitude of factors including water stabilization, reduced disturbance by large herbivores and reduced frequency of fire. *Phragmites australis* has the ability to thrive under such disturbance as a result of its ecophysiological strategies, broad ecological amplitude, high evolutionary potential and high phenotypic plasticity (Eller and Brix 2012; Kettenring and Mock 2012; Kettenring *et al.* 2015). *Phragmites australis* populations in southern Africa belong to the MED lineage, and compared to other lineages respond most positively to eutrophication due to high phenotypic plasticity (Eller and Brix 2012; Eller *et al.* 2017). Therefore, increasing nutrient loads can result in reeds having increased biomass production, shoot density (Romero *et al.* 1999; Tho *et al.* 2016), inflorescence and floret production (Kettenring *et al.* 2011) as well as seedling success (Kettenring *et al.* 2015). Reed expansion can also occur in *P. mauritianus* populations in southern Africa and reed encroachment is most often associated with changes to water flow and sedimentation (van Coller *et al.* 1997; Kotschy *et al.* 2000).

## Conclusions

A critical part of understanding the status of a plant as an introduced or native species is to have a clear understanding of its evolutionary history (Pyšek *et al.* 2004). However, understanding such history has often been based on poor data, inappropriate criteria or intuition (Pyšek 1995). The genetic results from this study suggest that both *P. australis* and *P. mauritianus* have had a long history in southern Africa and thus a cryptic invasion by non-native haplotypes has not occurred. Therefore, unlike in cryptic invasions it is the native populations that are expanding in their range and abundance. In light of this, management of reed encroachment should focus on addressing factors that may be contributing to expansion such as eutrophication and water stabilization while still ensuring the conservation of the species.

## Sources of Funding

This work was supported by the South African Research Chairs Initiative of the Department of Science and Technology and the National Research Foundation of South Africa.

## Contributions by the Authors

All authors conceived the ideas; K.C. collected the data, performed the laboratory work and analysed the results; C.L. led the *waxy* band laboratory work; all authors led the writing.

## Conflict of Interest

None declared.

## Supporting Information

The following additional information is available in the online version of this article—

**Table S1**. Sampling sites for collection of *Phragmites australis* genetic material including site location, province in South Africa and GPS coordinates. Membership probability (*Q*) assigns the percentage of shared ancestry for each *P. australis* sample, based on Bayesian clustering analysis of four microsatellite loci (PaGT4, PaGT8, PaGT9, PaGT22) with STRUCTURE (Pritchard *et al.* 2000). According to the Evanno method, two populations were inferred (pop 1—red; pop 2—green) **[see****Supporting Information—Fig. S1****]**. The presence or absence of *waxy* bands, *Waxy*100 and *Waxy*200, is shown, symbols +/− indicate presence/absence, where absent indicates no clear amplification of a *waxy* band. Genotypes were assigned based on shared alleles from the four microsatellite markers.

**Table S2**. Sampling sites for collection of *Phragmites mauritianus* genetic material including site location, province in South Africa and GPS coordinates. Membership probability (*Q*) assigns the percentage of shared ancestry for each *P. mauritianus* sample, based on Bayesian clustering analysis of four microsatellite loci (PaGT4, PaGT8, PaGT9, PaGT22) with STRUCTURE (Pritchard *et al.* 2000). According to the Evanno method, three populations were inferred (pop 1—orange, pop 2—yellow, pop 3—green) **[see****Supporting Information—Fig. S1****]**. The presence or absence of *waxy* bands, *Waxy*100 and *Waxy*200, is shown, symbols +/− indicate presence/absence, where absent indicates no clear amplification of a *waxy* band. Genotypes were assigned based on shared alleles from the four microsatellite markers.

**Table S3**. Primer sequences for *grass-waxy* analysis.

**Table S4**. Summary of allele amplification for *P. australis* and *P. mauritianus.*

**Table S5**. Comparison between the southern African lineages of *P. australis* and *P. mauritianus* and four North American and European lineages of *P. australis* from Saltonstall (2003) in microsatellites genetic traits. The comparison is based on four microsatellite loci (PaGT4, PaGT8, PaGT9, PaGT22) and includes *n*: number of samples genotyped; *A*_o_ observed number of alleles at loci; dominant phenotypes: dominant alleles, values in parentheses are the frequency of each phenotype; *H*_o_: observed heterozygosity.

**Figure S1**. Graphs of Delta *K* values showing the ideal number of populations (*k*) using four microsatellite primer pairs and the Evanno method implemented in STRUCTURE HARVESTER program according to Earl and vonHoldt (2012). (a) *k* = 2 based on 55 populations of *P. australis* and *P. mauritianus* in southern Africa, (b) *k* = 2 based on 39 populations of *P. australis* in South Africa, (c) *k* = 3 based on 16 populations of *P. mauritianus* in South Africa.

## Supplementary Material

Supplementary Material

## References

[CIT0001] Ailstock MS, Center E 2000 Adaptive strategies of common reed *Phragmites australis*. In: Proceedings: the role of phragmites in the Mid-Atlantic Region. A Management Symposium, Richmond, VA, 1–7.

[CIT0002] Baldwin AH, Kettenring KM, Whigham DF 2010 Seeds banks of *Phragmites australis*-dominated wetlands: relationships to seed viability, inundation, and land cover. Aquatic Botany 93:163–169.

[CIT0003] Brix H 1999 The European research project on reed die-back and progression (EUREED). Limnology Ecology and Management of Inland Waters 29:5–10.

[CIT0004] Canavan K, Paterson ID, Hill MP 2014 The herbivorous arthropods associated with the invasive alien plant, *Arundo donax*, and the native analagous plant, *Phragmites australis* in the Free State Province, South Africa. African Entomology 22:454–459.

[CIT0005] Chambers RM, Meyerson LA, Saltonstall K 1999 Expansion of *Phragmites australis* into tidal wetlands of North America. Aquatic Botany 64:261–273.

[CIT0006] Chu H, Cho WK, Jo Y, Kim W, Rim Y, Kim J 2011 Identification of natural hybrids in Korean *Phragmites* using haplotype and genotype analyses. Plant Systematics and Evolution 293:247.

[CIT0007] Clevering OA, Lissner J 1999 Taxonomy, chromosome numbers, clonal diversity and population dynamics of *Phragmites australis*.Aquatic Botany 64:185–208.

[CIT0008] Cowling RM, Hilton-Taylor C 1997 Phytogeography, flora and endemism. In: Cowling RM, Richardson DM, Pierce SM, eds. Vegetation of Southern Africa. Cambridge: Cambridge University Press, 43–56.

[CIT0009] Cronk JK, Fennessy MS 2016 Wetland plants: biology and ecology. Boca Raton, FL: CRC Press.

[CIT0010] Dlugosch KM, Parker IM 2008 Founding events in species invasions: genetic variation, adaptive evolution, and the role of multiple introductions. Molecular Ecology 17:431–449. 17908213 10.1111/j.1365-294X.2007.03538.x

[CIT0011] Driver A, Nel JL, Snaddon K, Murray K, Roux DJ, Hill L, Swartz ER, Manuel J, Funke N 2011 Implementation manual for freshwater ecosystem priority areas. WRC Report No. 1801/1/11.

[CIT0012] Earl DA, vonHoldt BM 2012 STRUCTURE HARVESTER: a website and program for visualizing STRUCTURE output and implementing the Evanno *method*.Conservation Genetics Resources 4:359–361.

[CIT0013] Eller F, Brix H 2012 Different genotypes of *Phragmites australis* show distinct phenotypic plasticity in response to nutrient availability and temperature. Aquatic Botany 103:89–97.

[CIT0014] Eller F, Skálová H, Caplan JS, Bhattarai GP, Burger MK, Cronin JT, Guo WY, Guo X, Hazelton ELG, Kettenring KM, Lambertini C, McCormick MK, Meyerson LA, Mozdzer TJ, Pyšek P, Sorrell BK, Whigham DF, Brix H 2017 Cosmopolitan species as models for ecophysiological responses to global change: the common reed *Phragmites australis*. Frontiers in Plant Science 8:1833. 29250081 10.3389/fpls.2017.01833 PMC5715336

[CIT0015] Evanno G, Regnaut S, Goudet J 2005 Detecting the number of clusters of individuals using the software structure: a simulation study. Molecular Ecology 14:2611–2620. 15969739 10.1111/j.1365-294X.2005.02553.x

[CIT0016] Fanshawe DB 1972 The biology of the reed - *Phragmites mauritianus* Kunth. Kirkia 8:147–150.

[CIT0017] Fernandes M, Adams J 2016 Quantifying the loss of and changes in estuary habitats in the uMkhomazi and Mvoti estuaries, South Africa. South African Journal of Botany 107:179–187.

[CIT0018] Gao L, Tang S, Zhuge L, Nie M, Zhu Z, Li B, Yang J 2012 Spatial genetic structure in natural populations of *Phragmites australis* in a mosaic of saline habitats in the yellow river delta, China. PLoS One 7:e43334. 22916244 10.1371/journal.pone.0043334 PMC3420903

[CIT0019] Gordon-Gray KD, Ward CJ 1971 A contribution to knowledge of *Phragmites* (Gramineae) in South Africa, with particular reference to Natal populations. South African Journal of Botany 37:1–30.

[CIT0020] Guo WY, Lambertini C, Li XZ, Meyerson LA, Brix H 2013 Invasion of Old World *Phragmites australis* in the New World: precipitation and temperature patterns combined with human influences redesign the invasive niche. Global Change Biology 19:3406–3422. 23765641 10.1111/gcb.12295

[CIT0022] Hazelton ELG, Mozdzer TJ, Burdick DM, Kettenring KM, Whigham DF 2014 *Phragmites australis* management in the United States: 40 years of methods and outcomes. AoB Plants 6:plu001. 24790122 10.1093/aobpla/plu001 PMC4038441

[CIT0023] Hellings SE, Gallagher JL 1992 The effects of salinity and flooding on *Phragmites australis*. Journal of Applied Ecology 29:41–49.

[CIT0024] Kearse M, Moir R, Wilson A, Stones-Havas S, Cheung M, Sturrock S, Buxton S, Cooper A, Markowitz S, Duran C, Thierer T, Ashton B, Meintjes P, Drummond A 2012 Geneious Basic: an integrated and extendable desktop software platform for the organization and analysis of sequence data. Bioinformatics 28:1647–1649. 22543367 10.1093/bioinformatics/bts199 PMC3371832

[CIT0025a] Kettenring KM, McCormick MK, Baron HM, Whigham DF 2011 Mechanisms of *Phragmites australis* invasion: feedbacks among genetic diversity, nutrients, and sexual reproduction. Journal of Applied Ecology 48:1305–13.

[CIT0025] Kettenring KM, Mock KE 2012 Genetic diversity, reproductive mode, and dispersal differ between the cryptic invader, *Phragmites australis*, and its native conspecific. Biological Invasions 14:2489–2504.

[CIT0026] Kettenring KM, Whigham DF, Hazelton EL, Gallagher SK, Weiner HM 2015 Biotic resistance, disturbance, and mode of colonization impact the invasion of a widespread, introduced wetland grass. Ecological Applications 25:466–480. 26263668 10.1890/14-0434.1

[CIT0027] Kotschy KA, Rogers KH, Carter AJ 2000 Patterns of change in reed cover and distribution in a seasonal riverine wetland in South Africa. Folia Geobotanica 35:363–373.

[CIT0028] Kumar S, Stecher G, Peterson D, Tamura K 2012 MEGA-CC: computing core of molecular evolutionary genetics analysis program for automated and iterative data analysis. Bioinformatics 28:2685–2686. 22923298 10.1093/bioinformatics/bts507 PMC3467750

[CIT0029] Lambert AM, Dudley TL, Saltonstall K 2010 Ecology and impacts of the large-statured invasive grasses *Arundo donax* and *Phragmites australis* in North America. Invasive Plant Science and Management 3:489–494.

[CIT0030] Lambertini C 2016 Heteroplasmy due to chloroplast paternal leakage: another insight into *Phragmites* haplotypic diversity in North America. Biological Invasions 18:2443–2455.

[CIT0031] Lambertini C, Eller FP, Achenbach L, Loc NX, Guo W-Y, Brix H 2012a Revisiting *Phragmites australis* variation in the Danube Delta with DNA molecular techniques. In: Gâştescu P, Lewis W, Breţcan P, eds. Water resources and wetlands. Tulcea, Romania, 142–150.

[CIT0032] Lambertini C, Gustafsson MHG, Frydenberg J, Lissner J, Speranza M, Brix H 2006 A phylogeographic study of the cosmopolitan genus *Phragmites* (Poaceae) based on AFLPs. Plant Systematics and Evolution 258:161–182.

[CIT0033] Lambertini C, Mendelssohn IA, Gustafsson MH, Olesen B, Riis T, Sorrell BK, Brix H 2012a Tracing the origin of gulf coast *Phragmites* (Poaceae): a story of long-distance dispersal and hybridization. American Journal of Botany 99:538–551. 22334449 10.3732/ajb.1100396

[CIT0034] Lambertini C, Sorrell BK, Riis T, Olesen B, Brix H 2012b Exploring the borders of European *Phragmites* within a cosmopolitan genus. AoB Plants 2012:pls020. 22962631 10.1093/aobpla/pls020 PMC3435523

[CIT0035] Lelong B, Lavoie C, Jodoin Y, Belzile F 2007 Expansion pathways of the exotic common reed (*Phragmites australis*): a historical and genetic analysis. Diversity and Distributions 13:430–437.

[CIT0036] Lemley DA, Adams JB, Strydom NA 2017 Testing the efficacy of an estuarine eutrophic condition index: does it account for shifts in flow conditions?Ecological Indicators 74:357–370.

[CIT0037] Lewontin RC 1995 The apportionment of human diversity. In: Dobzhansky T, Max K. Hecht MK, Steere WC, eds. Evolutionary biology. Chicago, IL: Springer, 381–398.

[CIT0038] Loveless MD, Hamrick JL 1984 Ecological determinants of genetic structure in plant populations. Annual Review of Ecology and Systematics 15:65–95.

[CIT0039] Mason-Gamer RJ, Weil CF, Kellogg EA 1998 Granule-bound starch synthase: structure, function, and phylogenetic utility. Molecular Biology and Evolution 15:1658–1673. 9866201 10.1093/oxfordjournals.molbev.a025893

[CIT0040] McKee J, Richards A 1996 Variation in seed production and germinability in common reed (*Phragmites australis*) in Britain and France with respect to climate. New Phytologist 133:233–243. 10.1111/j.1469-8137.1996.tb01890.x 29681068

[CIT0041] Meyerson LA, Cronin JT, Pyšek P 2016 *Phragmites* as a model system. Biological Invasions 18:2421–2431.

[CIT0042] Meyerson LA, Lambertini C, McCormick MK, Whigham DF 2012 Hybridization of common reed in North America? The answer is blowing in the wind. AoB Plants 2012:pls022. 22993684 10.1093/aobpla/pls022 PMC3444738

[CIT0043] Nei M 1973 Analysis of gene diversity in subdivided populations. Proceedings of the National Academy of Sciences of the United States of America 70:3321–3323. 4519626 10.1073/pnas.70.12.3321 PMC427228

[CIT0044] Peakall R, Smouse PE 2012 GenAlEx 6.5: genetic analysis in excel. Population genetic software for teaching and research–an update. Bioinformatics 28:2537–2539. 22820204 10.1093/bioinformatics/bts460 PMC3463245

[CIT0045] Pritchard JK, Stephens M, Donnelly P 2000 Inference of population structure using multilocus genotype data. Genetics 155:945–959. 10835412 10.1093/genetics/155.2.945 PMC1461096

[CIT0046] Pyšek P 1995 On the terminology used in plant invasion studies. In: Pyšek P, Prach K, Rejmanek M, Wade M, eds. Plant invasions: general aspects and special problems. Amsterdam, The Netherlands: SPB Academic Publishing, 71–81.

[CIT0047] Pyšek P, Richardson DM, Pergl J, Jarosík V, Sixtová Z, Weber E 2008 Geographical and taxonomic biases in invasion ecology. Trends in Ecology & Evolution 23:237–244. 18367291 10.1016/j.tree.2008.02.002

[CIT0048] Pyšek P, Richardson DM, Rejmánek M, Webster GL, Williamson M, Kirschner J 2004 Alien plants in checklists and floras: towards better communication between taxonomists and ecologists. Taxon 53:131–143.

[CIT0049] Richardson DM, Holmes PM, Esler KJ, Galatowitsch SM, Stromberg JC, Kirkman SP, Pyšek P, Hobbs RJ 2007 Riparian vegetation: degradation, alien plant invasions, and restoration prospects. Diversity and Distributions 13:126–139.

[CIT0052a] Romero JA, Brix H, Comín FA 1999 Interactive effects of N and P on growth, nutrient allocation and NH_4_ uptake kinetics by Phragmites australis. Aquatic Botany 64:369–380.

[CIT0050] Russell IA 2003 Long-term changes in the distribution of emergent aquatic plants in a brackish South African estuarine-lake system. African Journal of Aquatic Science 28:103–122.

[CIT0051] Russell IA, Kraaij T 2008 Effects of cutting *Phragmites australis* along an inundation gradient, with implications for managing reed encroachment in a South African estuarine lake system. Wetlands Ecology and Management 16:383–393.

[CIT0052] Rutherford MC, Mucina L, Powrie LW 2006 Biomes and bioregions of Southern Africa. In: The vegetation of South Africa, Lesotho and Swaziland. Pretoria: South African National Biodiversity Institute, 30–51.

[CIT0053] Saltonstall K 2002 Cryptic invasion by a non-native genotype of the common reed, *Phragmites australis*, into North America. Proceedings of the National Academy of Sciences of the United States of America 99:2445–2449. 11854535 10.1073/pnas.032477999 PMC122384

[CIT0054] Saltonstall K 2003 Microsatellite variation within and among North American lineages of *Phragmites australis*. Molecular Ecology 12:1689–1702. 12803624 10.1046/j.1365-294x.2003.01849.x

[CIT0055] Saltonstall K, Lambert A, Meyerson LA 2010 Genetics and reproduction of common (*Phragmites australis*) and giant reed (*Arundo donax*). Invasive Plant Science and Management 3:495–505.

[CIT0056] Schulze RE, McGee OS 1978 Climatic indices and classification in relation to the biogeography of Southern Africa. In: Werger MJA, ed. Biogeography and ecology of Southern Africa. Monographiae Biologicae, Vol. 31 Dordrecht: Springer.

[CIT0057] Scott L 1982 A late quaternary pollen record from Transvaal bushveld, South Africa. Quaternary Research 17:339–370.

[CIT0058] Sieben EJJ, Collins NB, Kotze DC, Mofutsanyana SS, Janks M 2017 Temperate grassy wetlands of South Africa: description, classification and explanatory environmental factors. South African Journal of Botany 113:68–76.

[CIT0059] Takahashi R, Nishio T, Ichizen N, Takano T 2007 Salt-tolerant reed plants contain lower Na+ and higher K+ than salt-sensitive reed plants. Acta Physiologiae Plantarum 29:431–438.

[CIT0060] te Beest M, Le Roux JJ, Richardson DM, Brysting AK, Suda J, Kubesová M, Pysek P 2011 The more the better? The role of polyploidy in facilitating plant invasions. Annals of Botany 109:19–45. 22040744 10.1093/aob/mcr277 PMC3241594

[CIT0061a] Tho BT Sorrell BK Lambertini C Eller F Brix H 2016 Phragmites australis: How do genotypes of different phylogeographic origins differ from their invasive genotypes in growth, nitrogen allocation and gas exchange?Biological invasions 18:2563–76.

[CIT0061] Tscharntke T 1992 Fragmentation of *Phragmites* habitats, minimum viable population size, habitat suitability, and local extinction of moths, midges, flies, aphids, and birds. Conservation Biology 6:530–536.

[CIT0062] Turesson G 1922 The species and the variety as ecological units. Hereditas 3:100–113.

[CIT0064] Van Coller A, Rogers K, Heritage G 1997 Linking riparian vegetation types and fluvial geomorphology along the Sabie River within the Kruger National Park, South Africa. African Journal of Ecology 35:194–212.

[CIT0065] Veldkamp JF 1994 Miscellaneous notes on Southeast-Asian Gramineae .9. Setaria and Paspalidium. Blumea 39:373–384.

[CIT0066] Waisel Y 1972 Biology of halophytes. New York: Academic Press.

[CIT0067] Weisser PJ, Parsons RJ 1981 Monitoring *Phragmites australis* increases from 1937 to 1976 in the Siyai Lagoon (Natal, South Africa) by means of air photo interpretation. Bothalia 13:553–556.

[CIT0068] Wu CA, Lowry DB, Cooley AM, Wright KM, Lee YW, Willis JH 2008 Mimulus is an emerging model system for the integration of ecological and genomic studies. Heredity 100:220–230. 17551519 10.1038/sj.hdy.6801018

[CIT0069] Yeh FC, Boyle J 1997 POPGENE, the user-friendly shareware for population genetic analysis. Molecular Biology and Biotechnology 434:724–731.

