# Supplementary material for: Expansive reed populations—alien invasion or disturbed wetlands?
Source: AoB Plants. 2018 Feb 23;10(2):ply014. doi: 10.1093/aobpla/ply014 (PMC5861408; doi:10.1093/aobpla/ply014)
Supplement: Supplementary Material [file ply014_suppl_supplementary-material.docx]

## SUPPORTING INFORMATION

**Table S1** Sampling sites for collection of *P. australis* genetic material including site location, province in South Africa and GPS coordinates. Membership probability (Q) assigns the percentage of shared ancestry for each *P. australis* sample, based on Bayesian clustering analysis of four microsatellite loci (PaGT4, PaGT8, PaGT9, PaGT22) with STRUCTURE (Pritchard *et al.* 2000). According to the Evanno method, two populations were inferred (pop 1 - red, pop 2 - green) (Fig. S6). The presence or absence of waxy bands, *Waxy*100 and *Waxy*200 is shown, symbols +/- indicate presence/absence, where absent indicates no clear amplification of a *waxy* band. Allele genotype were assigned based on shared alleles from the four microsatellite markers.

| **Sample number** | **Site Location** | **Province** | **GPS coordinates** | **Membership probability (Q)**  **(population average)** | | **Waxy 100** | **Waxy 200** | **Allele**  **genotype** |
| --- | --- | --- | --- | --- | --- | --- | --- | --- |
|  |  |  |  | **Pop 1** | **Pop 2** |  |  |  |
| 1,2,3,7,45,46,47 | Port St. John’s | Eastern Cape | 31°35’58.11’’S, 29°31’55.51’’E | 0.988 | 0.012 | + | + | A |
| 9,41,42 | Kosi Bay | KwaZulu-Natal | 26°57’39.46’’S, 32°49’36.23’’E | 0.973 | 0.027 | + | + | B, C |
| 37 | Durban | KwaZulu-Natal | 29°48’31.72’’S, 31°01’05.94’’E | 0.515 | 0.485 | + | + | F |
| 38 | Durban | KwaZulu-Natal | 29°48’31.72’’S, 31°01’05.94’’E | 0.515 | 0.485 | + |  | F |
| 43,44 | St Lucia | KwaZulu-Natal | 28°22’10.82’’S, 32°24’35.84’’E | 0.983 | 0.017 | + | + | D, E |
| 53 | Golden Gate Nature Reserve | Free State | 28°29’58.51’’S,  32°49’09’’E | 0.029 | 0.971 | - | + | G |
| 59 | Hartebeespoort Dam | North  West | 25°44’22.37’’S,  27°54’18.77E | 0.218 | 0.782 | - | + | H |
| 61 | Stellenbosch | Western Cape | 33°58’06’’S,  18°47’27’’E | 0.012 | 0.998 |  |  | I |
| 62 | Knysna | Western Cape | 34°02’33’’S,  23°04’01’’E | 0.024 | 0.976 | + | - | J |
| 63 | Kars River | Western Cape |  |  |  | + | + | K |
| 64 | Rocherpan Nature Reserve | Western Cape | 33°40’16.28’’S,  26°38’36.78’’E | 0.059 | 0.941 | + | + | L |
| 65 | Cape Point | Western Cape | 34°14’28’’S,  18°25’28’’E | 0.114 | 0.886 | - | + | M |
| 66 | Bushman’s River | Eastern Cape | 33°40’16.28’’S,  26°38’36.78’’E | 0.033 | 0.967 |  |  | N |
| 68 | Plettenberg | Western Cape | 34°04’04’’S,  23°21’45’’E | 0.331 | 0.669 |  |  | 0 |
| 69 | Bot River | Western Cape | 34°18’47.06’’S, 19°08’54.69’’E | 0.022 | 0.978 | + | + | P |
| 70 | Buffels River | Northern Cape | 33°11’44.66’’S, 20°51’09.60’’E | 0.014 | 0.986 | + | - | Q |
| 71 | Somerset West | Western Cape | 34°05’25.63’’S, 18°50’15.37’’E | 0.625 | 0.375 | + | - | R |
| 72 | Berg River | Western Cape | 33°52’39.84’’S, 19°02’02.38’’E | 0.015 | 0.985 |  |  | S |
| 73 | Theewaterskloof | Western Cape | 34°04’11’’S,  19°17’41’’E | 0.184 | 0.816 |  |  | T |
| 74 | Marquard | Free State | 28°42’05.56’’S, 27°26’33.14’’E | 0.035 | 0.965 | - | + | U |
| 75 | Baviaans River | Western Cape | 33°58’24’’S,  23°38’51’’E | 0.036 | 0.964 | - | + | V |
| 76 | Ladybrand | Free State | 29°10’59.60’’S, 27°28’25.85’’E | 0.169 | 0.831 | + | + | W |
| 77 | Wilderness Lagoon | Western Cape | 29°10’59.60’’S, 22°34’58.33’’E | 0.020 | 0.980 |  |  | X |
| 78 | Agulhas National Park | Western Cape | 34°44’33.32’’S, 19°39’25.66’’E | 0.029 | 0.971 |  |  | Y |
| 79, 97 | Clocolan | Free State | 28°51’30.10’’S, 27°32’27.38’’E | 0.574 | 0.426 | - | + | Z |
| 80 | Marine Drive | Western Cape | 33°52’42.96’’S, 18°29’25.56’’E | 0.021 | 0.979 |  |  | AA |
| 81 | Alexandria | Eastern Cape | 33°38’01’’S,  26°19’07’’E | 0.053 | 0.947 | - | + | AB |
| 82 | Ratelfontein | Western  Cape | 31°30’34.66’’S, 23°37’46.26’’E | 0.036 | 0.964 |  |  | AC |
| 83 | Seekoei | Northern Cape | 31°38’01’’S,  26°19’07’’E | 0.034 | 0.966 |  |  | AD |
| 84 | Eland’s Bay | Western Cape | 32°18’52.89’’S, 18°21’19.79’’E | 0.392 | 0.608 | - | + | AE |
| 85 | La Mercy | KwaZulu-Natal | 29°38’47’’S;  31°07’18’’E | 0.984 | 0.016 |  |  | AF |
| 86 | Zimbali | KwaZulu-Natal | 29°33’38’’S;  31°10’19’’E | 0.313 | 0.687 |  |  | AG |
| 87,88,89,90,91 | Ballito | KwaZulu-Natal | 29°28’17’’S;  31°14’28’’E | 0.974 | 0.026 | - | + | AH |
| 92,93,94,95,96 | Clocolan | Free State | 28°51’30.10’’S, 27°32’27.38’’E | 0.014 | 0.986 |  |  | AI |
| 98 | Hout Bay | Western Cape | 34°02’05.64’’S; 18°21’13.93’’E | 0.009 | 0.991 | + | + | AK |
| 99,100,101,102,103 | Constantia | Western Cape | 34°01’24.92’’S; 18°26’18.85’’E | 0.054 | 0.946 |  |  | AL |
| 104 | Jonkershoek | Western Cape | 33°58’33.98’’S; 18°56’40.24’E | 0.037 | 0.963 | - | + | AM |
| 105 | Spier Estate | Western Cape | 33°58’30.82’’S;  18°47’07.84”E | 0.039 | 0.961 | - | + | AN |
| 106 | Mulderbosch | Western Cape | 33°56’51.80’’S; 18°45’55.06”E | 0.021 | 0.979 |  |  | AO |

**Table S2** Sampling sites for collection of *P. mauritianus* genetic material including site location, province in South Africa and GPS coordinates. Membership probability (Q) assigns the percentage of shared ancestry for each *Phragmites mauritianus* sample, based on Bayesian clustering analysis of four microsatellite loci (PaGT4, PaGT8, PaGT9, PaGT22) with STRUCTURE (Pritchard *et al.* 2000). According to the Evanno method, three populations were inferred (pop 1 - orange, pop 2 – yellow, pop 3 - green) (Fig. S6). The presence or absence of waxy bands, *Waxy*100 and *Waxy*200 is shown, symbols +/- indicate presence/absence, where absent indicates no clear amplification of a *waxy* band. Allele genotype were assigned based on shared alleles from the four microsatellite markers.

| **Sample**  **number** | **Site Location** | **Province** | **GPS coordinates** | **Membership probability**  **(population average)** | | | ***Waxy* 100** | ***Waxy* 200** | **Allele genotype** |
| --- | --- | --- | --- | --- | --- | --- | --- | --- | --- |
|  |  |  |  | **Pop 1** | **Pop 2** | **Pop 3** |  |  |  |
| 1M | Little Gowrie | Mpumalanga | 24°40’51’’S, 31°33’17.60’’E | 0.111 | 0.521 | 0.368 | + | - | A |
| 6M | Little Gowrie | Mpumalanga | 24°40’51’’S, 31°33’17.60’’E | 0.020 | 0.051 | 0.929 | + | - | B |
| 7M | Little Gowrie | Mpumalanga | 24°40’51’’S, 31°33’17.60’’E | 0.048 | 0.831 | 0.121 | + | - | C |
| 2M,8M,9M | Mbabane | Swaziland | 26°19’15.54’’S, 31°07’36.60’’E | 0.015 | 0.023 | 0.962 | + | - | D, E |
| 3M,10M,11M,14M | Umhlanga | KwaZulu-Natal | 29°42’49.80’’S, 31°05’31.23’’E | 0.978 | 0.012 | 0.010 | + | - | F |
| 12M | Umhlanga | KwaZulu-Natal | 29°42’49.80’’S, 31°05’31.23’’E | 0.414 | 0.072 | 0.513 | + | - | G |
| 4M | Sozisa | Swaziland | 26°19’42.24’’S, 31°08;34.78’’E | 0.013 | 0.758 | 0.228 | + | - | H |
| 5M, 13M | Sozisa | Swaziland | 26°19’42.24’’S, 31°08;34.78’’E | 0.015 | 0.235 | 0.750 | + | - | I |
| 15M,16M,  17M,18M,  19M,31M | Shakaskraal | KwaZulu-Natal | 29°27’28’’S; 31°13’00’’E | 0.017 | 0.956 | 0.027 | + | - | J, K |
| 20M | Zimbali | KwaZulu-Natal | 29°33’38’’S; 31°10’19’’E | 0.096 | 0.847 | 0.057 | + | - | L |
| 21M | Umgeni River | KwaZulu-Natal | 29°47’48’’S; 30°57’57’’E | 0.845 | 0.093 | 0.062 | + | - | M |
| 22M | Nelspruit | Mpumalanga | 25°26’03.57’’S; 30°52’02.49’’E | 0.075 | 0.588 | 0.337 | + | - | - |
| 23M,33M,34M38M | Nelspruit | Mpumalanga | 25°01’52.73’’S;31°01’25.43’’E | 0.845 | 0.112 | 0.043 | + | - | N, O, P |
| 35M, 36M, 37M | Nelspruit | Mpumalanga | 25°01’52.73’’S; 31°01’25.43’’E | 0.030 | 0.607 | 0.363 | + | - | Q |
| 24M | Hazyview | Mpumalanga | 25°27’17.05’’S; 30°53’07.36’’E | 0.597 | 0.354 | 0.050 | + | - | R |
| 39M | Hazyview | Mpumalanga | 25°27’17.05’’S; 30°53’07.36’’E | 0.231 | 0.742 | 0.027 | + | - | S |
| 25M, 40M | Sabie Sun-Hazyview | Mpumalanga | 25°02’10.75’’S; 31°07’05.83’’E | 0.024 | 0.884 | 0.093 | + | - | T |
| 26M, 27M,28M,29M30M | Kruger National Park | Mpumalanga | 24°58’48.04’’S; 31°28’53.93’’E | 0.974 | 0.015 | 0.011 | + | - | U, V |
| 41M | Kruger National Park | Mpumalanga | 24°58’48.04’’S; 31°28’53.93’’E | 0.036 | 0.938 | 0.027 | + | - | W |
| 32M | Nelspruit – Hippo Lodge | Mpumalanga | 25°26’06.83’’S; 30°52’04.82’’E | 0.413 | 0.491 | 0.096 | + | - | X |
| 42M | Ngalwana Village | Zambia | 14°59’76.90’’S; 23°11’29.70’’E | 0.334 | 0.544 | 0.122 | + | - | Y |
| 43M | Barotse Floodplains | Zambia | 15°51’18,30’’S; 23°13’42.00’’E | 0.066 | 0.845 | 0.089 | + | - | Z |
| 44M | Lungwebungu Mouth | Zambia | 14°30’77,30’’S; 23°18’76.70’’E | 0.251 | 0.652 | 0.096 | + | - | AA |

Table S3 Primer sequences for *grass-waxy* analysis.

| **Primer** | **Sequence** | **Length of**  **sequence** |
| --- | --- | --- |
| WaxyF^1^ | 5’ TGCGAGCTCGACAACATCATGCG | 200 bp |
| Waxy200(25)R^1^ | 5’ GATCCCTCGCCATCACARCATCGCC |  |
| Waxy100F^2^ | 5´- CGATAGGATGAGCAGTTAGG | 100 bp |
| WaxyR | 5´- GGCGAGCGGCGCGATCCCTCGCC |  |

^1^Mason-Gamer *et al.* 1998; ^2^Lambertini *et al*. 2012 *c*

Table S4 Summary of allele amplification for *P. australis* and *P. mauritianus.*

|  | ***P. australis*** | ***P. mauritianus*** |
| --- | --- | --- |
| Number of samples (n) | 61 | 44 |
| Total number of alleles | 36 | 27 |
| Mean number of alleles per sample | 2.16 ± 0.94 | 1.56 ± 0.71 |
| 1 allele amplified (%) | 27.27% | 44% |
| 2 alleles amplified (%) | 34.54% | 56% |
| 3 alleles amplified (%) | 33.20% | 0% |
| 4 alleles amplified (%) | 5% | 0% |

Table S5 Comparison between the southern African lineages of *P. australis* and *P. mauritianus* and four North American and European lineages of *P. australis* from Saltonstall (2003) in microsatellites genetic traits. The comparison is based on four microsatellite loci (PaGT4, PaGT8, PaGT9, PaGT22) and includes *n*: Number of samples genotyped; A_o_ observed number of alleles at loci; Dominant phenotypes: dominant alleles, values in parentheses are the frequency of each phenotype; *H_o_*: observed heterozygosity.

| **Locus** | **Haplotype Lineage** | | | | | | |  |
| --- | --- | --- | --- | --- | --- | --- | --- | --- |
|  | **Introduced** | **Native** | **Gulf Coast** | | | **Europe** | ***P. australis***  **South Africa** | ***P. mauritianus***  **South Africa** |
| **All Loci** | | | | | | | |  |
| Mean A_0_ | 7 ± 3.56 | 5.5 ± 3 | | 2.67 ± 0.58 | 8.5 ± 4.20 | | 9 ± 2.83 | 6.75 ± 1.26 |
| Mean *H*_0_ | 0.43 ± 0.18 | 0.14 ± 0.12 | | 0.96 ± 0.05 | 0.52 ± 0.24 | | 0.77 ± 0.22 | 0.55 ± 0.13 |
| ***PaGT4*** | | | | | | | |  |
| *n* | 150 | 125 | | 21 | 57 | | 58 | 43 |
| A_0_ | 9 | 3 | | 3 | 6 | | 7 | 7 |
| Dominant phenotypes | 274 (0.12)  276 (0.43)  274/276 (0.30) | 266 (0.73)  274 (0.16) | | 274/276/280 (0.86) | 274 (.40)  276 (0.11)  274/276 (0.19) | | 272/274/276 (0.26)  274/276 (0.26) | 270 (0.35)  270/278 (0.18) |
| *H*_0_ | 0.40 | 0.10 | | 0.90 | 0.33 | | 0.88 | 0.42 |
| ***PaGT8*** |  |  | |  |  | |  |  |
| *n* | 131 | 92 | | 22 | 57 | | 56 | 42 |
| A_0_ | 2 | 3 | | 2 | 4 | | 7 | 7 |
| Dominant phenotypes | 176 (0.76)  176/178 (0.19) | 178 (0.71)  180 (0.14) | | 176/189 (0.95) | 176 (0.49)  178 (0.18)  176/178 (0.25) | | 173/175/180 (0.13)  175/180/182 (0.2) | 177/180 (0.19)  177/184 (0.19) |
| *H*_0_ | 0.19 | 0.04 | | 0.95 | 0.33 | | 0.80 | 0.72 |
| ***PaGT9*** |  |  | |  |  | |  |  |
| *n* | 121 | 109 | | 21 | 45 | | 57 | 44 |
| A_0_ | 10 | 9 | | 3 | 13 | | 13 | 5 |
| Dominant phenotypes | 198 (0.34)  198/202 (0.24) | 210 (0.62)  210/212 (0.20) | | 192/196 (0.95) | 198/204 (0.09)  198/206 (0.11) | | 192/200/231 (0.11)  200/202/215 (0.1) | 200 (0.34)  196/200 (0.16) |
| *H*_0_ | 0.55 | 0.31 | | 1 | 0.83 | | 0.95 | 0.52 |
| ***PaGT22*** |  |  | |  |  | |  |  |
| *n* | 95 | 25 | | 7 | 36 | | 61 | 35 |
| A_0_ | 7 | 7 | | 3 | 11 | | 9 | 8 |
| Dominant phenotypes | 181 (0.20)  197 (0.13)  181/193 (0.21)  181/197 (0.18) | 185 (0.24)  191 (0.40)  193 (0.12) | | 175/183/197 (1.00) | 183 (0.08)  195 (0.14)  199 (0.08)  195/199 (0.08) | | 183 (0.52)  183/202 (0.24) | 183 (0.27)  188 (0.12)  175/183 (0.18) |
| *H*_0_ | 0.58 | 0.12 | | 1 | 0.58 | | 0.46 | 0.53 |

| a   | b   | c   |
| --- | --- | --- |

**Figure S6.** Graphs of Delta K values showing the ideal number of populations (*k*) using four microsatellite primer pairs and the Evanno method implemented in STRUCTURE HARVESTER program according to Earl and von Holdt (2012). a. *k* = 2 based on 55 populations of *P. australis* and *P. mauritianus* in Southern Africa, b. *k* = 2 based on 39 populations of *P. australis* in South Africa, c. *k* = 3 based on 16 populations of *P. mauritianus* in Southern Africa.
